# Artificial Intelligence-Based Prediction of Pancreatic Stone Clearance in Pancreatolithiasis Using Pretreatment CT Images and Clinical Features

**DOI:** 10.3390/diagnostics16172700

**Published:** 2026-08-24

**Authors:** Satoshi Yamamoto, Atsushi Teramoto, Tomoyuki Ono, Senju Hashimoto, Yoshiaki Katano, Takashi Kobayashi, Hisanori Muto, Yoshihiko Tachi, Hironao Miyoshi, Kazuo Inui

**Affiliations:** 1 Department of Gastroenterology, Fujita Health University Bantane Hospital, Nagoya 454-8509, Japan; syama@fujita-hu.ac.jp (S.Y.);; 2 Graduate School of Science and Engineering, Meijo University, Nagoya 468-8502, Japan; 3 Department of Radiological Technology, Fujita Health University Bantane Hospital, Nagoya 454-8509, Japan; 4 Department of Gastroenterology, Fujita Health University Okazaki Medical Center, Okazaki 444-0829, Japan; 5 Yuki Geriatric Health Services Facility, Handa 475-0828, Japan; 6 Department of Gastroenterology, Yamashita Hospital, Ichinomiya 491-8531, Japan

**Keywords:** pancreatolithiasis, chronic pancreatitis, extracorporeal shock wave lithotripsy, computed tomography, artificial intelligence, deep learning

## Abstract

**Background/Objectives:** Nonsurgical treatment for pancreatolithiasis is widely performed. However, treatment success remains difficult to predict before treatment initiation, complicating the selection of an appropriate treatment strategy. This study aimed to predict pancreatic stone clearance after nonsurgical treatment for pancreatolithiasis associated with chronic pancreatitis by integrating pretreatment computed tomography (CT) images and clinical information using deep learning and machine learning models. **Methods:** Of 195 patients with pancreatolithiasis associated with chronic pancreatitis who underwent nonsurgical treatment, including extracorporeal shock wave lithotripsy, at our institution between 1992 and 2024, only 91 (47%) had extractable pretreatment noncontrast abdominal CT images and were included in the AI analysis. Multiple deep learning models (VGG16/19, InceptionV3, ResNet50, DenseNet121/169/201, Vision Transformer, and Swin Transformer) were trained using CT images, and their predictive performance was compared. Imaging-derived and clinical predictors selected using only the training data in each patient-level cross-validation fold were combined and used as inputs for conventional machine learning models, including random forest, support vector machine, naïve Bayes, neural network, and gradient boosting. **Results:** Successful pancreatic stone clearance was achieved in 54 of 91 patients (59%). Compared with the 104 patients without extractable CT data, the analyzed cohort had a higher proportion of asymptomatic pancreatolithiasis (43% vs. 15%) and a markedly lower pancreatic stone clearance rate (59% vs. 88%), indicating potential selection bias. Asymptomatic pancreatolithiasis and a pancreatic stone size of ≥15 mm, defined using a data-derived exploratory cutoff, were significantly associated with unsuccessful stone clearance. Among the deep learning models, ResNet50 achieved the highest performance (area under the receiver operating characteristic curve [AUC], 0.718), followed by Vision Transformer (Large model, 16 × 16 patches) (AUC, 0.700). When image-derived features were combined with clinical features, the neural network achieved the best performance, with a mean AUC of 0.757, a median sensitivity of 0.568, a median specificity of 0.704, and a median accuracy of 0.659. **Conclusions:** A neural network integrating CT-derived image features with clinical information showed moderate internal predictive performance for pancreatic stone clearance. Because this was a single-center retrospective study without external validation, the present model should be regarded as a preliminary predictive model requiring validation in independent cohorts before clinical application.

## 1. Introduction

Treatment guidelines for chronic pancreatitis with pancreatolithiasis have been published by academic medical societies in Japan and Western countries [1,2,3,4,5,6]. As chronic pancreatitis progresses, pancreatic stones may form within the main pancreatic duct. Pancreatic duct stones can impair pancreatic juice outflow and increase intraductal pancreatic pressure, resulting in abdominal pain and recurrent acute exacerbations, and may contribute to complications such as pancreatic pseudocysts. Repeated inflammation may further promote progression of chronic pancreatitis and lead to deterioration of both endocrine and exocrine pancreatic function. Therefore, removal of pancreatic stones in symptomatic patients is clinically important because it can improve pancreatic juice drainage, relieve pain, and may reduce recurrent acute exacerbations [1,2,3,4,5]. In Japan, endoscopic treatment including extracorporeal shock wave lithotripsy (ESWL) is generally performed as first-line treatment for symptomatic pancreatic stones, with surgical treatment often reserved for difficult cases [2]. The effectiveness of nonsurgical treatment with ESWL and/or endoscopic treatment has also been reported in several studies and meta-analyses, including recent international reports [7,8,9,10,11,12,13]. However, a certain proportion of patients fail to achieve stone clearance despite undergoing multiple ESWL sessions. In this context, the authors conducted a retrospective study in 2024 to determine an appropriate number of ESWL sessions for pancreatic stone treatment and concluded that either medical management, including the use of pain relievers, or surgical treatment should be considered instead of further ESWL in patients requiring eight or more ESWL sessions [14]. A complicating problem is that the effectiveness of ESWL for an individual patient cannot be determined until treatment has actually been performed, which places an additional burden on patients. In the field of urolithiasis, CT-based radiomics, machine learning, and deep learning approaches have already been applied to predict stone-free status after ESWL, and several studies have reported favorable predictive performance [15]. Thus, the general concept of predicting ESWL outcomes using pretreatment CT images and AI-based methods is not new. However, these studies have focused on urinary tract stones, and their findings cannot be directly extrapolated to pancreatolithiasis associated with chronic pancreatitis. In pancreatolithiasis, several studies have evaluated conventional quantitative CT parameters, including mean stone density (MSD) measured in Hounsfield units (HU), as potential predictors of stone clearance and other treatment outcomes after ESWL [16,17,18,19,20]. Nevertheless, pancreatic stone clearance is a multifactorial outcome that may be influenced by clinical, anatomical, and procedural factors beyond stone density alone. To our knowledge, the present study is the first to apply pretreatment CT-based AI to the prediction of pancreatic stone clearance after nonsurgical treatment for pancreatolithiasis.

In recent years, medical image analysis using deep learning has advanced rapidly, and its application to diagnostic support and prognostic prediction across a wide range of medical imaging modalities has been reported [21]. In the area of pancreatology, the utility of artificial intelligence (AI) for diagnostic support, such as the identification of pancreatic cancer on CT images, has been reported [22,23]. As that example illustrates, most AI studies have focused on diagnosis. Fewer studies have used AI to predict treatment outcomes using imaging data, such as analyzing pretreatment CT to determine likelihood of successful ESWL for pancreatolithiasis. Among deep learning architectures, convolutional neural networks (CNNs) [21,24] and transformer-based models, including Vision Transformer (ViT), have been increasingly applied to medical image analysis [25].

Therefore, the aim of this study was to develop and internally evaluate a preliminary predictive model for pancreatic stone clearance after nonsurgical treatment for pancreatolithiasis associated with chronic pancreatitis, using pretreatment CT images and clinical information. The novelty of this study lies in applying CT-based AI prediction to pancreatolithiasis rather than in proposing a new AI methodology itself.

## 2. Materials and Methods

The study was conducted in accordance with the Declaration of Helsinki and was originally approved by the Ethical Review Committee of Fujita Health University (approval no. HM23-254) on 16 October 2023. An amendment to the study protocol, including an extension of the study period and revision of the planned sample size, was subsequently approved by the same committee (approval no. HM26-271) on 4 August 2026.

**Figure 1:**
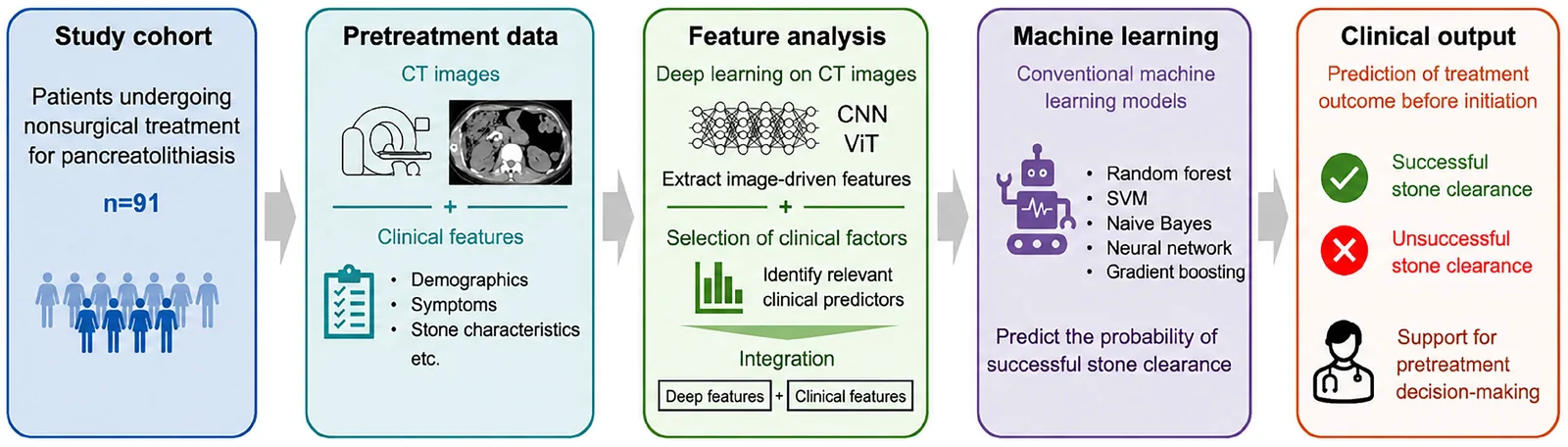
Overview of the proposed AI-based framework for predicting pancreatic stone clearance outcomes.

An overview of the overall analytical workflow and model construction is shown in Figure 1. Between 1992 and 2024, a total of 195 patients with pancreatolithiasis associated with chronic pancreatitis underwent nonsurgical treatment including ESWL at our institution. Among them, 91 patients whose pretreatment noncontrast abdominal CT image data could be extracted were included in the analysis. Eligibility for the AI analysis was determined solely by the availability of extractable pretreatment noncontrast abdominal CT image data suitable for image processing; no selection based on clinical characteristics or treatment outcomes was performed. The pretreatment CT images used for the AI analysis were acquired between December 2006 and 2024. In older cases, archived CT image data were no longer available because they had been deleted in accordance with the institutional data retention policy.

Multiple deep learning models, including VGG16, VGG19, Inception V3, ResNet50, DenseNet121, DenseNet169, DenseNet201, Vision Transformer (Base model, 16 × 16 patches) (ViT-B/16), Vision Transformer (Large model, 16 × 16 patches) (ViT-L/16), and Swin Transformer base and large models, were trained using CT images from patients with pancreatolithiasis who underwent ESWL with or without endoscopic treatment. Predictive performances of the models concerning pancreatic stone clearance outcomes were then comparatively evaluated. Next, imaging-derived and clinical predictors selected within the training data of each cross-validation fold were combined for subsequent machine learning analysis. These integrated feature sets were used to train several machine learning models, including random forest, support vector machine, Naive Bayes, neural network, and gradient boosting, and their performance in predicting treatment outcomes was compared across models.

Pretreatment CT images and clinical features were analyzed using deep learning and machine learning models to predict successful or unsuccessful stone clearance before treatment initiation.

Pancreatic stones were diagnosed with CT imaging. To confirm that stones were located within the main pancreatic duct rather than in the pancreatic parenchyma or branch ducts, endoscopic retrograde cholangiopancreatography (ERCP) was performed in all patients. The median observation period was 13 months (range, 0–206). Median patient age was 61 years (range, 22–83), and the male:female ratio was 6:1 (78 men and 13 women). Etiologies of chronic pancreatitis were alcoholic in 54 patients (59%) and nonalcoholic in 37 (41%). Stones were solitary in 41 patients (45%) and multiple in 50 (55%). Mean stone diameter was 13 mm (standard deviation, 5). For each patient, stone distribution within the main pancreatic duct was localized to the pancreatic head, body, and/or tail. Boundaries of these segments were defined according to the TNM classification [26]. Seventy-six patients (84%) had stones in 1 segment; 15 (16%) had stones in 2 segments or more. Thirteen patients (14%) had a stricture of the main pancreatic duct, defined as localized marked stenosis (diameter less than 2 mm) with dilation of the duct proximal to the stenosis (Table 1). In patients with impacted stones, presence of ductal stricture was assessed using magnetic resonance cholangiopancreatographic images performed after stone clearance, or findings from ERCP performed to remove stone fragments. We found pancreatolithiasis accompanied by pain to be the usual indication for nonsurgical treatment [6,7,8,9,10,11,13]. Additional indications included interventions to preserve or improve pancreatic function or observation of main pancreatic duct dilation behind an impacted pancreatic stone.

All patients underwent ESWL. Some also required endoscopic pancreatic sphincterotomy or endoscopic minor papilla sphincterotomy to allow natural expulsion of a stone. We performed endoscopic stone removal with a basket catheter when other measures could not remove stones. Successful treatment of pancreatolithiasis was defined as disappearance of pancreatic stones from the main pancreatic duct. Stone clearance was assessed using follow-up CT and, in most cases, ERCP, to confirm the absence of residual stones in the main pancreatic duct. Treatment outcome was evaluated at the time when disappearance of stones from the main pancreatic duct was confirmed, or when further nonsurgical treatment was judged to be ineffective because stone fragmentation and clearance had reached a plateau despite repeated treatment. In the latter situation, the case was classified as unsuccessful stone clearance. These assessments were made by the attending pancreatobiliary physicians in routine clinical practice. Because this was a retrospective real-world clinical study, outcome assessment was not blinded. This endpoint represented post-treatment clearance of stones from the main pancreatic duct and did not represent durable pain relief or recurrence-free ductal clearance. A follow-up period of 0 months indicated that no additional long-term observation was available at our institution after treatment response assessment, rather than absence of post-treatment outcome assessment. Our ESWL device was an electrohydraulic spark gap system (Tripter X-1; Direx Medical Systems, Ltd., Petah Tikva, Israel). The same ESWL device model was used throughout the entire clinical study period from 1992 to 2024, and the basic treatment strategy for pancreatolithiasis remained unchanged during this period. The approximate total number of shocks delivered ranged from 2000 to 3000 per ESWL session.

**Figure 2:**
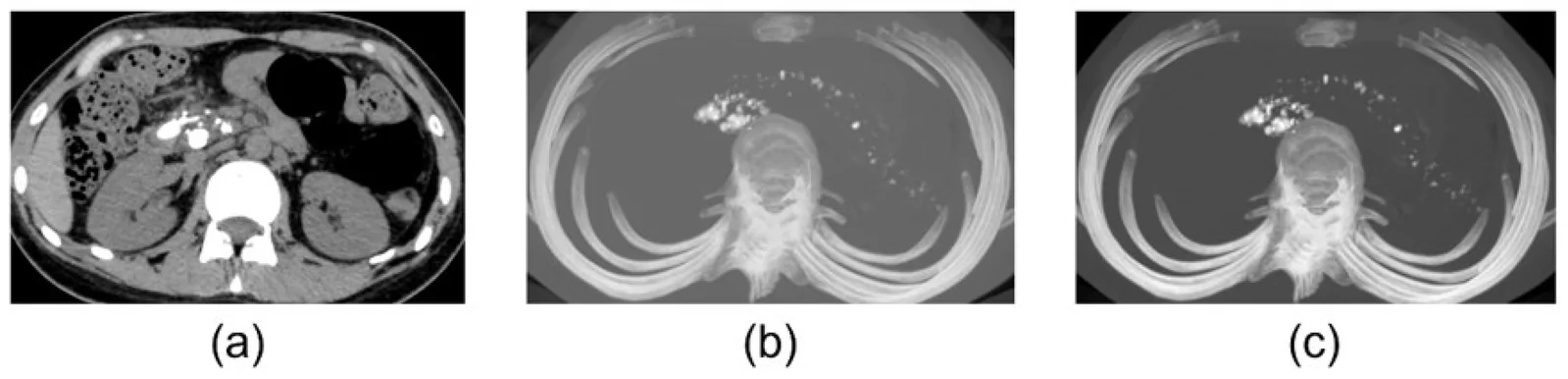
CT image preprocessing for deep learning analysis. (**a**) Original axial noncontrast abdominal CT image in the soft tissue window setting. (**b**) Maximum-intensity projection (MIP) image generated along the *z*-axis from the CT volume data. (**c**) Bone window image after window conversion (window level, 500; window width, 2000), enhancing visibility of pancreatic stones.

For image data extraction, DICOM-format CT images displaying the entire pancreas along the *z*-axis were obtained for each patient. The z-axis range containing the entire pancreas was manually defined using ImageJ (version 1.54g; National Institutes of Health, Bethesda, MD, USA).CT equipment and acquisition parameters differed among patients. To reduce contamination from non-target calcifications and other high-attenuation structures, the CT volume used for MIP generation was intentionally restricted to the slices containing the pancreas along the *z*-axis. Nevertheless, these preprocessing steps could not completely eliminate heterogeneity related to CT equipment and acquisition conditions, which may have affected the image features learned by the models. Based on these images, maximum-intensity projections were generated along the *z*-axis. The maximum-intensity projection images were then converted to a bone window setting (window level, 500; window width, 2000) and saved in 8-bit PNG format (Figure 2). This image-processing strategy was used to emphasize calcified pancreatic stones on noncontrast CT while preserving their anatomical relationship with the pancreas. Because pancreatic stones appear as high-attenuation structures, maximum-intensity projection images were used to visualize calcified lesions distributed along the pancreatic duct. The bone window setting was applied to improve visualization of calcified stones and reduce the influence of surrounding soft-tissue density. By including the entire pancreas along the *z*-axis, information on stone distribution, multiplicity, and location within the pancreatic head, body, and tail could be retained. This method also enabled the generation of standardized two-dimensional images from three-dimensional CT datasets for use across multiple deep learning models. Manual segmentation of the pancreas or pancreatic duct was not performed because the pancreas has a complex and highly variable contour, which may be further altered by atrophy and morphological changes associated with chronic pancreatitis. Consequently, accurate manual segmentation is time-consuming and susceptible to operator-dependent variability, and reliable automated segmentation may also be technically challenging in such cases. This preprocessing workflow was designed to generate standardized two-dimensional MIP images for deep learning analysis and was not intended to preserve quantitative CT attenuation values such as HU density measured from the original DICOM data.

For deep learning model development, seven convolutional neural network (CNN) models and four ViT-based models were employed. CNN architectures included VGG16/19, Inception V3, ResNet50 [27], and DenseNet121/169/201 [28]. These CNN backbones and ImageNet-based transfer learning strategies have been widely used in medical image classification and computer-aided detection [29,30]. ViT-based architectures included the ViT-B/16, the ViT-L/16 [25], and the Swin Transformer base and large models [31]. All deep learning models were implemented using TensorFlow (version 2.10; Google LLC, Mountain View, CA, USA) with the Keras API. The ViT-B/16 and ViT-L/16 models were implemented using vit-keras (version 0.1.2), while the Swin Transformer models and their ImageNet-pretrained weights were obtained from the Swin-Transformer-TF implementation available on GitHub (Swin-Transformer-TF, GitHub repository, accessed on 29 April 2025). All CNN and ViT models were initialized with weights pretrained on ImageNet, a large-scale natural image dataset. The final fully connected layers were replaced with task-specific layers consisting of 1024 units followed by 2 output units, and the models were fine-tuned as a binary classification task to discriminate between successful and unsuccessful pancreatic stone clearance. During fine-tuning, models were trained for 100 epochs with a batch size of 16 and a learning rate of 1 × 10^−5^, using the Adam optimizer. Unsuccessful pancreatic stone clearance was defined as the positive class. The comparison among the deep learning architectures was conducted as an exploratory analysis and was not intended to provide definitive evidence of the superiority of a particular architecture. Model performance was evaluated using 5-fold cross-validation. During cross-validation, images from the same patient were not assigned to both the training and test sets. In each fold, the ImageNet-pretrained models were fine-tuned using only the training data, and model performance was evaluated using the corresponding held-out data. Image features were extracted from the fine-tuned models within the same cross-validation procedure. Predictive performance was evaluated using area under the receiver operating characteristic curve (AUC), sensitivity, specificity, and accuracy. For model interpretability, Gradient-weighted Class Activation Mapping (Grad-CAM) [32] was applied to CNN models to visualize regions contributing to predictions, while Attention Maps were used for ViT-based models to visualize attention regions during inference.

To identify clinical features influencing success or failure of stone clearance, differences in patient characteristics, including presence of symptoms, etiology, number of stones, size of stones, distribution of stones, and presence of main pancreatic duct stricture, were compared between cases with successful and unsuccessful pancreatic stone clearance. Categorical variables were analyzed using the chi-square test, and multivariate analysis was performed using logistic regression. A *p* value of less than 0.05 was considered statistically significant. In addition, receiver operating characteristic (ROC) curve analysis was performed to determine the pancreatic stone diameter associated with unsuccessful stone clearance attempts. The area under the curve (AUC) was calculated, and the cutoff value was defined as the point that maximized the Youden index (sensitivity + specificity − 1). In the present cohort, 15 mm was identified as a data-derived exploratory cutoff value; therefore, maximum pancreatic stone size was categorized as ≤14 mm or ≥15 mm. Using this cutoff, a forced-entry multivariable logistic regression analysis was performed with unsuccessful pancreatic stone clearance as the dependent variable and symptom status and pancreatic stone size (≤14 mm vs. ≥15 mm) as independent variables. These whole-cohort analyses were performed to evaluate clinical factors associated with pancreatic stone clearance and were not used for predictor selection in the subsequent machine learning analysis. For the machine learning analysis, maximum pancreatic stone size was treated as a continuous variable. To assess potential selection bias, available clinical characteristics and stone clearance outcomes were compared between patients with and without extractable pretreatment noncontrast abdominal CT image data. Statistical analyses of clinical variables were performed using IBM SPSS Statistics (version 28.0.1.0; IBM Corp., Armonk, NY, USA).

For the integrated analysis using machine learning models, predictor selection was performed independently within the training dataset of each patient-level cross-validation fold. Among the 11 deep learning models, the two imaging-derived predictors with the highest univariate AUC values in the training dataset were selected. Among the candidate clinical variables, the two predictors with the smallest *p* values in univariate logistic regression within the training dataset were selected. The held-out test fold was not used for predictor selection. These combined features were then entered into commonly used supervised machine learning models for clinical prediction, including random forest, support vector machine, Naive Bayes, neural network, and gradient boosting, to predict the success or failure of stone clearance attempts [33,34,35]. All machine learning analyses were performed using Orange (version 3.39; University of Ljubljana, Ljubljana, Slovenia). Unsuccessful clearance attempts were defined as the positive class. Patient-level stratified 5-fold cross-validation was used for internal evaluation. The complete training and evaluation workflow was executed five times using the same cross-validation fold assignment to assess run-to-run variability, with different random seeds used for stochastic model training. One overall AUC value was obtained from each execution, and the mean and SD across the five AUC values were calculated. Sensitivity, specificity, and accuracy were summarized as the median and IQR across the five executions. The completed CLAIM and TRIPOD-AI checklists are provided as File S1 and File S2, respectively.

## 3. Results

### 3.1. Treatment Results and Comparison of Pancreatic Stone Clearance Rates Based on Clinical Features

Among the 91 patients whose noncontrast abdominal CT images were available, 54 (59%) achieved successful stone clearance. The median number of ESWL sessions was 6 (range, 1–61); the number of sessions was unavailable for one patient. The patient who underwent 61 ESWL sessions was treated over a prolonged period before the stopping criterion proposed in our previous study was established; therefore, this exceptionally high number does not represent our current treatment strategy [14]. Receiver operating characteristic curve analysis identified a data-derived exploratory cutoff of 15 mm for pancreatic stone size. In comparisons of stone clearance rates based on clinical features among the 91 patients, no significant differences were associated with age, sex, etiology, number of stones, stone distribution, or presence of a main pancreatic duct stricture. However, the clearance rate was significantly lower in patients who had asymptomatic pancreatolithiasis (46%, 18/39) compared with those with symptomatic pancreatolithiasis (69%, 36/52; *p* = 0.045) (Table 2). Further, the pancreatic stone clearance rate was significantly lower in patients with a stone size of 15 mm or greater (32%, 9/28) than in those with a stone size of 14 mm or less (71%, 45/63; *p* = 0.001) (Table 2). In a forced-entry multivariable logistic regression analysis including symptom status and pancreatic stone size, asymptomatic pancreatolithiasis (adjusted OR, 2.820; 95% CI, 1.105–7.191; *p* = 0.030) and pancreatic stone size ≥15 mm (adjusted OR, 5.550; 95% CI, 2.042–15.085; *p* < 0.001) were independently associated with unsuccessful pancreatic stone clearance. A plain logistic regression model using symptom status and pancreatic stone size achieved an AUC of 0.738 (95% CI, 0.632–0.844). To assess potential selection bias, patients with and without extractable pretreatment noncontrast abdominal CT image data were compared (Supplementary Table S1). Significant differences were observed in symptom status, the presence of main pancreatic duct stricture, and the stone clearance rate, whereas maximum pancreatic stone size did not differ significantly between the two groups. Patients with extractable CT data had a higher proportion of asymptomatic pancreatolithiasis (43% vs. 15%) and a lower stone clearance rate (59% vs. 88%) than those without extractable CT data.

### 3.2. Performance Comparison Among Deep Learning Models

**Figure 3:**
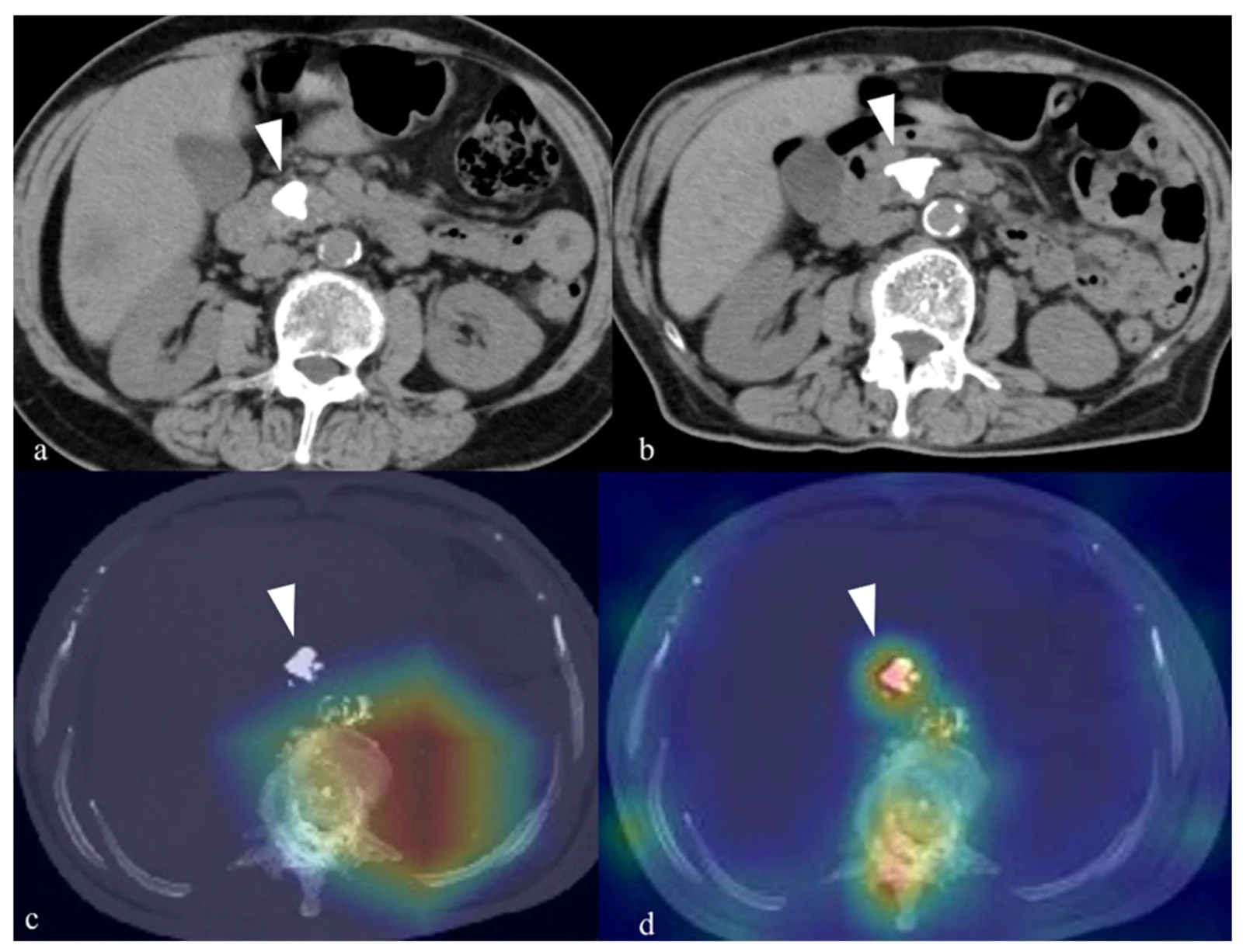
A case of unsuccessful pancreatic stone clearance following nonsurgical treatment. (**a**) Pretreatment CT image of a patient with asymptomatic idiopathic chronic pancreatitis. A 20-mm pancreatic stone is present in the main pancreatic duct within the pancreatic head (arrowhead). (**b**) Post-treatment CT image. After a total of 19 ESWL sessions, the stone in the pancreatic head remained (arrowhead), representing unsuccessful stone clearance. ResNet50 predicted unsuccessful pancreatic stone clearance with a probability of 62%, while the ViT-L/16 predicted unsuccessful clearance with a probability of 61%. (**c**) Grad-CAM visualization of a ResNet50. The highlighted region does not correspond to the pancreatic stone (arrowhead), indicating that the model attended to a different region during prediction. (**d**) Attention Map visualization of the ViT-L/16. The highlighted region corresponds appropriately to the pancreatic stone (arrowhead), indicating that the model attended to the anatomically relevant region.

When classification performance of the deep learning models was compared, ResNet50 demonstrated the highest predictive performance with an AUC of 0.718, followed by ViT-L/16 with an AUC of 0.700 (Table 3). However, the highest AUC among these models was 0.718, indicating only moderate and clinically suboptimal discrimination. Visualization of regions of interest during prediction indicated that ViT-L/16 focused on anatomically relevant regions (Figure 3).

### 3.3. Comparison of Predictive Performance Among Machine Learning Models

Predictor selection was performed independently within each training dataset. ResNet50-derived predictions and stone size were selected in all five folds, whereas ViT-L/16-derived predictions and symptom status were selected in four of the five folds. In the remaining fold, DenseNet121-derived predictions and etiology were selected. Using these fold-specific predictor sets, the neural network model demonstrated the highest predictive performance, with a mean AUC of 0.757 (SD, 0.039), a median sensitivity of 0.568 (IQR, 0.541–0.595), a median specificity of 0.704 (IQR, 0.685–0.741), and a median accuracy of 0.659 (IQR, 0.626–0.670) (Table 4). When prediction was performed using only the two imaging-derived predictors selected within each training fold, the neural network achieved the best performance, with a mean AUC of 0.739 (SD, 0.016), a median sensitivity of 0.486 (IQR, 0.486–0.514), a median specificity of 0.759 (IQR, 0.685–0.796), and a median accuracy of 0.648 (IQR, 0.615–0.670). When prediction was performed using only the two clinical predictors selected within each training fold, logistic regression achieved the highest mean AUC of 0.702 (SD, 0.000), with a median sensitivity of 0.405 (IQR, 0.405–0.405), a median specificity of 0.759 (IQR, 0.759–0.759), and a median accuracy of 0.615 (IQR, 0.615–0.615).

## 4. Discussion

The principal finding of this study is that a preliminary AI-based model integrating pretreatment CT images and clinical information showed moderate internal performance in predicting pancreatic stone clearance after nonsurgical treatment for pancreatolithiasis. This study should be regarded as an initial application of CT-based AI prediction to pancreatolithiasis rather than as a novel AI methodology.

In chronic pancreatitis, the formation of pancreatic stones can obstruct pancreatic juice outflow, leading to increased intraductal pressure and consequent pain. These changes may accelerate fibrosis of the pancreatic parenchyma, progressively impairing both exocrine and endocrine pancreatic function. Because removal of pancreatic stones improves pancreatic juice outflow, it may reduce pain and slow the progression of chronic pancreatitis. Nonsurgical treatment, including ESWL with or without endoscopic therapy, is recommended as first-line treatment for pancreatolithiasis. When nonsurgical treatment is unsuccessful, transition to conservative management with oral medications, including analgesics, may be considered to alleviate symptoms. Surgical treatment should be considered according to various guidelines when severe pain persists [1,2,3,4,5]. Nonsurgical treatment for pancreatolithiasis, including ESWL, has been widely used as a less invasive treatment option for pancreatic duct stones [36]. Previous clinical studies and meta-analyses have reported variable pancreatic stone clearance rates after ESWL with or without endoscopic therapy [7,8,9,10,11,12,13]. In the meta-analysis cited as reference [11], the pooled complete ductal clearance rate was 69.8% (95% CI, 63.8–75.5%). In the present study, the stone clearance rate after ESWL with or without endoscopic therapy was 59%, which was 10.8 percentage points lower than the pooled estimate. However, this comparison should be interpreted cautiously because patient characteristics, patient selection criteria, and the types of ESWL devices used may have differed among studies. Regarding clinical factors associated with pancreatic stone clearance, previous studies have reported that larger stones are associated with lower clearance rates [12]. Consistent with these findings, the present study demonstrated a significantly lower stone clearance rate in patients with a stone diameter of ≥15 mm. Furthermore, we previously reported [37] that the pancreatic stone clearance rate after ESWL with or without endoscopic treatment was significantly lower in patients with asymptomatic pancreatolithiasis than in those with symptomatic pancreatolithiasis (63% vs. 84%), whereas the stone fragmentation rate did not differ significantly between the two groups. This finding suggests that insufficient stone clearance after fragmentation in asymptomatic cases may be related to reduced pancreatic juice flow. Similarly, this study found the pancreatic stone clearance rate to be significantly lower in asymptomatic pancreatolithiasis than in symptomatic pancreatolithiasis. Therefore, the associations of pancreatic stone size and symptom status with stone clearance observed in the present study should be interpreted as confirmatory findings that replicate prior observations, rather than as novel clinical predictors unique to this cohort.

One major issue in the management of pancreatolithiasis is the difficulty in accurately predicting, before treatment initiation, whether nonsurgical treatment will be successful. As a result, a certain proportion of patients fail to achieve complete stone clearance despite undergoing multiple ESWL sessions. In our previous study [14], we suggested that either medical management, including the use of pain relievers, or surgical treatment should be considered instead of further ESWL in patients requiring eight or more ESWL sessions. From the perspective of patient burden, the ability to predict unsuccessful stone clearance prior to treatment initiation would allow avoidance of excessively repeated nonsurgical interventions including ESWL, facilitating earlier transition to alternative therapeutic strategies. The AI-based predictive model developed in the present study integrates pretreatment CT imaging and clinical information, enabling prediction of treatment outcomes prior to intervention. This approach may improve treatment selection and support informed consent discussions before initiation of nonsurgical treatment for pancreatolithiasis. In urolithiasis, CT-based radiomics and machine learning models have been used to predict stone-free status after ESWL, with reported AUCs generally higher than those observed in the present study. Therefore, the present results should not be interpreted as showing performance comparable to mature urolithiasis models. In the present study, the best-performing deep learning model achieved an internal AUC of 0.718, and the integrated model combining image-derived features with clinical features achieved an internal AUC of 0.757. These values indicate moderate internal predictive performance and should be interpreted cautiously because external validation was not performed.

An additional limitation of the present study is that quantitative CT attenuation of pancreatic stones was not evaluated. Several studies using noncontrast CT have reported associations between pancreatic stone density, measured in Hounsfield units (HU), and stone clearance and other treatment outcomes after ESWL [16,17,18,19,20]. In the study by Liu et al., MSD was significantly associated with stone clearance, with an optimal cutoff value of 1000.45 HU, a sensitivity of 78.0%, a specificity of 48.6%, and an AUC of 0.6373. Although these findings suggest that CT attenuation may provide potentially useful quantitative information, the reported discriminatory performance was modest, and its independent predictive value and optimal cutoff remain insufficiently established.

Prediction of nonsurgical treatment outcomes in pancreatolithiasis is likely to be multifactorial. Unlike urinary tract stones, pancreatic stones occur in the setting of chronic pancreatitis, and stone clearance may be influenced not only by stone density but also by stone size and number, main pancreatic duct stricture and anatomy, pancreatic juice drainage, endoscopic accessibility, papillary intervention, feasibility of endoscopic stone extraction, and chronic inflammatory changes. In the present study, *z*-axis maximum intensity projection reconstruction, fixed bone-window settings, and conversion to 8-bit PNG images were used for image preprocessing. Although this workflow may retain visual information related to stone morphology and contrast, it does not preserve the original quantitative HU values from the DICOM data. Therefore, the present model should not be interpreted as a replacement for HU-based quantitative assessment. Rather, it should be regarded as a preliminary attempt to integrate morphological and visual information derived from standardized pretreatment CT images with clinically relevant factors to address a multifactorial prediction task.

In the clinical application of deep learning models, it is important not only to evaluate predictive performance but also to visualize the basis of the model’s decision-making and assess its validity. In this study, Grad-CAM visualizations of ResNet50 did not consistently demonstrate attention to regions corresponding to pancreatic stones, while the Attention Maps of ViT-L/16 did. Although ResNet50 demonstrated relatively favorable predictive performance, the discordance between its predictive performance and the Grad-CAM findings should be interpreted cautiously. The model may have partially relied on background image characteristics or acquisition-related factors rather than stone-specific features, and the possibility of shortcut learning cannot be excluded. Although manual segmentation was avoided to reduce processing time and operator-dependent variability, the use of unsegmented images retained surrounding non-target structures, which may have increased the risk of shortcut learning. Future studies should evaluate whether standardized or automated localization or segmentation can reduce this risk while maintaining reproducibility.

Another important finding of the present study is that the integration of clinical information with image-derived features resulted in a numerically higher predictive performance than that of the image-feature-only models. In particular, the integrated neural network achieved a mean AUC of 0.757 (SD, 0.039), compared with a mean AUC of 0.739 (SD, 0.016) for the image-feature-only neural network. As noted above, symptom status and pancreatic stone size were associated with stone clearance in the whole-cohort clinical analysis, consistent with prior studies, and should therefore be regarded as confirmatory clinical findings rather than novel predictors identified in this study. The main significance of the present study lies in integrating clinical features with AI-derived image features from pretreatment CT images to construct a preliminary predictive model for pancreatic stone clearance. These results suggest that factors not fully captured by CT-based morphologic information alone contribute to prediction of pancreatic stone clearance. Specifically, clinical features such as symptom status and pancreatic stone size may influence treatment outcomes through mechanisms independent of image-derived features. By integrating image features with clinical features, the model was able to learn multidimensional aspects of treatment outcomes that cannot be fully captured by a single modality. The integrated AI model developed in the present study may enable pretreatment prediction of outcomes of nonsurgical treatment for pancreatolithiasis associated with chronic pancreatitis, and might assist in treatment decision-making and informed consent discussions prior to pancreatic stone treatment. However, the predictive performance of this model was evaluated only by repeated patient-level 5-fold cross-validation within the same 91-patient cohort. Given the limited sample size relative to the capacities of the deep learning architectures evaluated, overfitting cannot be completely excluded. Therefore, the present model should be regarded as a preliminary predictive model requiring independent hold-out validation and external validation before clinical application. In addition, the model achieved a median sensitivity of 0.568 for detecting unsuccessful stone clearance, indicating that a substantial proportion of eventual treatment failures would not be identified. Therefore, the present model should not be used as a stand-alone or definitive basis for individual treatment decisions, including whether to continue nonsurgical treatment or transition to surgery. It should also not be used as the sole basis for informed consent at this stage. External validation and further improvement in sensitivity are required before clinical application.

The improvement in predictive performance after combining image-derived features with clinical features is clinically reasonable. CT images provide morphological information, including stone burden, calcification pattern, pancreatic contour, and surrounding anatomical context. In contrast, clinical variables may reflect factors that cannot be fully assessed from CT images alone. In this study, symptom status and pancreatic stone size were identified as factors associated with stone clearance in the whole-cohort clinical analysis. These variables may be related to pancreatic juice flow, degree of ductal obstruction, and the clinical indication for treatment. Therefore, combining image features with clinical information may allow the model to evaluate treatment response from both anatomical and clinical perspectives. This point is important because the success of nonsurgical treatment for pancreatic stones is not determined only by the appearance of the stones on CT images. Even when stones appear similar on CT, differences in pancreatic juice drainage and clinical background may influence whether fragmented stones can be cleared from the main pancreatic duct. The present findings suggest that integrating imaging and clinical data may provide additional predictive information for pretreatment assessment beyond that provided by either image features or clinical variables alone. However, this apparent incremental value was not statistically confirmed because formal comparisons of AUCs, calibration analysis, and decision-curve analysis were not performed. Nevertheless, direct comparison between AI models for urolithiasis and the present model for pancreatolithiasis should be made cautiously. In urinary tract stone disease, post-ESWL stone-free status is more directly influenced by stone-related factors such as stone size, stone density, skin-to-stone distance, and anatomical location. In contrast, pancreatic stone clearance after nonsurgical treatment is affected not only by the stone itself but also by clinical, anatomical, and procedural factors specific to chronic pancreatitis, including main pancreatic duct stricture, pancreatic duct anatomy, pancreatic juice drainage, endoscopic accessibility, and feasibility of endoscopic stone extraction. Therefore, although urolithiasis studies provide an important methodological precedent, they should not be used as a direct performance benchmark for AI models predicting pancreatic stone clearance.

From a clinical standpoint, the proposed model may be particularly useful in patients for whom the expected benefit of repeated nonsurgical treatment is uncertain. In current practice, treatment strategies for pancreatic stones are often determined based on clinical information and the physician’s experience. However, these factors do not always allow accurate prediction of whether complete stone clearance can be achieved before treatment initiation. A pretreatment prediction model using routinely available CT images and clinical information could provide additional objective information when selecting between continued nonsurgical treatment, conservative management, or earlier consideration of surgical treatment. Such a model should not replace clinical judgment, but it may function as a decision-support tool to complement conventional assessment. In particular, when a patient is predicted to have a low probability of stone clearance, physicians may be able to explain the possibility of repeated unsuccessful ESWL sessions more clearly before treatment. Conversely, when a patient is predicted to have a favorable probability of stone clearance, nonsurgical treatment may be pursued with greater confidence. Therefore, AI-based pretreatment prediction may help clinicians select a more appropriate treatment strategy for individual patients with pancreatolithiasis. Previous studies of AI-assisted gastrointestinal endoscopy have shown that favorable performance observed under controlled research conditions may not necessarily translate into improved effectiveness in routine clinical practice [38]. Although the clinical task evaluated in the present study differs from computer-aided lesion detection during endoscopy, the same general caution regarding clinical translation applies. Therefore, internally validated predictive performance alone does not establish clinical utility, and external validation followed by prospective evaluation under routine clinical conditions is required before clinical application of the present model.

This study has several limitations. First, it was a single-center retrospective study. Second, sample size was limited. In addition, only patients with extractable pretreatment noncontrast abdominal CT image data were included in the AI analysis. Because older archived CT data had been deleted in accordance with the institutional data retention policy, CT data were preferentially available for more recent cases. Patients with extractable CT data had a higher proportion of asymptomatic pancreatolithiasis (43% vs. 15%) and a lower stone clearance rate (59% vs. 88%) than those without extractable CT data. In earlier treatment periods, patients were more commonly referred to our institution because of symptoms such as abdominal pain, whereas in recent years, asymptomatic patients with pancreatic stones detected on imaging have increasingly been referred for assessment of treatment indications. This change in referral patterns likely explains the higher proportion of asymptomatic patients among those with extractable CT data and was likely an important contributor to the lower stone clearance rate observed in this group. Therefore, the possibility of selection bias associated with the availability of pretreatment CT data cannot be excluded. Therefore, external validation using multicenter data is required to assess the generalizability of the present model. Another important limitation is that quantitative HU density of pancreatic stones was not evaluated. The present preprocessing workflow converted original CT data into standardized MIP images and 8-bit PNG format, and therefore quantitative attenuation information from the original DICOM data was not preserved. Because standardized ROI-based HU measurement was not included in the original analysis plan and was not performed uniformly in this retrospective cohort, HU density could not be added as a feature or baseline model in this revision. Future studies should evaluate HU density using standardized measurements from the original DICOM data and determine whether it provides incremental predictive value when combined with stone size, symptom status, and AI-derived image features. The use of MIP images with a fixed bone-window setting allowed calcified pancreatic stones to be represented in a standardized two-dimensional format without requiring manual segmentation of the pancreas or pancreatic stones. This approach reduced the preprocessing workload and operator dependence and allowed the same procedure to be applied consistently across patients. However, projecting volumetric CT data into a single two-dimensional MIP image inevitably compressed information along the *z*-axis. Consequently, detailed three-dimensional anatomical relationships among pancreatic stones, the pancreatic duct, and the pancreatic parenchyma may not have been fully preserved. Moreover, the model could not use the complete volumetric morphology and spatial distribution available in the original CT data. Thus, this preprocessing strategy involved a trade-off between practical reproducibility and the preservation of detailed three-dimensional anatomical information. A further limitation is that neither an independent hold-out test set nor an external validation cohort was available. Model performance was evaluated using repeated patient-level 5-fold cross-validation in 91 patients, with predictor selection performed independently within the training data of each fold. Therefore, the mean AUC of 0.757 achieved by the integrated neural network should be interpreted as an internal performance estimate and may be optimistic. Furthermore, formal comparisons of AUCs, calibration analysis, and decision-curve analysis were not performed. Therefore, the incremental value of the integrated model over the image-feature-only and clinical-feature-only models was not statistically confirmed, and its calibration performance and net benefit were not assessed. External validation using multicenter data is required to assess its generalizability.

## 5. Conclusions

In this single-center retrospective study, a neural network integrating deep learning-derived image features from pretreatment CT images with clinical information showed moderate internal predictive performance for pancreatic stone clearance after nonsurgical treatment for pancreatolithiasis associated with chronic pancreatitis. The main novelty of this study lies in the application of CT-based AI prediction to pancreatolithiasis rather than in the AI methodology itself. However, neither an independent hold-out test set nor an external validation cohort was available. Therefore, the present model should be regarded as a preliminary predictive model and should not be used as a stand-alone or definitive basis for individual treatment decisions, such as whether to continue nonsurgical treatment or transition to surgery, nor as the sole basis for informed consent at this stage. Further validation in independent multicenter cohorts is required before this approach can be applied as a clinical decision-support tool for nonsurgical treatment of pancreatolithiasis.

## Figures and Tables

**Table 1:** Characteristics of Chronic Pancreatitis.

Characteristic		Number of Patients (%)
Etiology		
	Alcoholic	54 (59)
	Non-alcoholic	37 (41)
Number of stones		
	Single	41 (45)
	Multiple	50 (55)
Size of Stones		
	≤14 mm	63 (69)
	≥15 mm	28 (31)
Distribution of Stones		
	One Area	76 (84)
	Two Areas or More	15 (16)
Main Pancreatic Duct Stricture		
	Present	13 (14)
	Absent	78 (86)

**Table 2:** Patient Characteristics and Stone Clearance Rate After Treatment (*n* = 91).

Characteristic		Stone Clearance Rate, %	*p*
Age			
	<65 (*n* = 56)	62	0.578
	≥65 (*n* = 35)	54	
Sex			
	Male (*n* = 78)	60	0.896
	Female (*n* = 13)	54	
Symptoms			
	Present (*n* = 52)	69	0.045
	Absent (*n* = 39)	46	
Etiology			
	Alcoholic (*n* = 54)	67	0.133
	Non-alcoholic (*n* = 37)	49	
Number of stones			
	Single (*n* = 41)	63	0.616
	Multiple (*n* = 50)	56	
Size of stones			
	≤14 mm (*n* = 63)	71	0.001
	≥15 mm (*n* = 28)	32	
Distribution of Stones			
	One area (*n* = 76)	58	0.730
	Two Areas or More (*n* = 15)	67	
Stricture of Main Pancreatic Duct			
	Present (*n* = 13)	62	1.000
	Absent (*n* = 78)	59	

**Table 3:** Performance of Deep Learning Models.

Model	Sensitivity	Specificity	Accuracy	AUC
VGG 16	0.459	0.685	0.593	0.634
VGG 19	0.595	0.574	0.582	0.636
Inception V3	0.595	0.574	0.582	0.616
ResNet 50	0.676	0.667	0.670	0.718
DenseNet121	0.703	0.611	0.648	0.645
DenseNet169	0.595	0.593	0.593	0.620
DenseNet201	0.649	0.574	0.604	0.619
Vision Transformer (Base model, 16 × 16 patches)	0.595	0.556	0.571	0.585
Vision Transformer (Large model, 16 × 16 patches)	0.784	0.593	0.670	0.700
Swin Transformer (Base model)	0.649	0.519	0.571	0.596
Swin Transformer (Large model)	0.649	0.648	0.648	0.659

Abbreviation: AUC, area under the receiver operating characteristic curve.

**Table 4:** Performance of conventional machine learning models.

Model	Median Sensitivity (IQR)	Median Specificity (IQR)	Median Accuracy (IQR)	Mean AUC (SD)
Random forest	0.514 (0.486–0.514)	0.704 (0.704–0.741)	0.615 (0.604–0.659)	0.728 (0.046)
Support vector machine	0.486 (0.486–0.486)	0.722 (0.722–0.722)	0.626 (0.626–0.626)	0.726 (0.024)
Naive Bayes	0.514 (0.514–0.514)	0.704 (0.704–0.704)	0.626 (0.626–0.626)	0.718 (0.024)
Neural network	0.568 (0.541–0.595)	0.704 (0.685–0.741)	0.659 (0.626–0.670)	0.757 (0.039)
Gradient boosting	0.486 (0.486–0.486)	0.667 (0.667–0.667)	0.593 (0.593–0.593)	0.697 (0.015)

Abbreviations: AUC, area under the receiver operating characteristic curve; SD, standard deviation; IQR, interquartile range. The complete patient-level stratified 5-fold cross-validation workflow was executed five times using the same fold assignment. The mean and SD were calculated across the five overall AUC values obtained from these executions. Sensitivity, specificity, and accuracy are presented as the median and IQR across the five executions. An SD of 0.000 indicates that the AUC values were identical across the five executions at the reported three-decimal precision. Identical lower and upper IQR limits indicate that the 25th and 75th percentiles were identical at the reported precision. These measures represent run-to-run variability and do not represent fold-to-fold variability, uncertainty associated with alternative patient partitions, or confidence intervals for model performance.

## Data Availability

The datasets presented in this article are not readily available due to privacy and ethical restrictions involving patient data. Requests to access the de-identified datasets should be directed to the corresponding author and will be considered after approval by the institutional ethics committee and completion of the required data-sharing procedures. The analysis code is available from the corresponding author upon reasonable request.

## Supplementary Materials

The following supporting information can be downloaded at: https://www.mdpi.com/article/10.3390/diagnostics16172700/s1, Table S1: Comparison of patient characteristics and stone clearance outcomes according to the availability of extractable pretreatment noncontrast abdominal CT image data; File S1: CLAIM Checklist; File S2: TRIPOD-AI Checklist.
