# Supplementary material for: Artificial Intelligence-Based Prediction of Pancreatic Stone Clearance in Pancreatolithiasis Using Pretreatment CT Images and Clinical Features
Source: Diagnostics (Basel). 2026 Aug 24;16(17):2700. doi: 10.3390/diagnostics16172700 (PMC13564669; doi:10.3390/diagnostics16172700)
Supplement: Supplementary file 1 [file diagnostics-16-02700-s001.zip › CLAIMChecklist_R2.pdf]

Checklist for Artificial Intelligence in Medical Imaging (CLAIM): 2024 Update

Section / Topic	No.	Item	Page / Line	No	NA
**TITLE / ABSTRACT**					
	**1**	Identification as a study of AI methodology, specifying the category of technology used (e.g., deep learning)	p. 1, lines 2–4, 18–21		
**ABSTRACT**					
	**2**	Summary of study design, methods, results, and conclusions	pp. 1–2, lines 16–46 (partially reported)		
**INTRODUCTION**					
	**3**	Scientific and/or clinical background, including the intended use and role of the AI approach	pp. 2–3, lines 51–95; p. 14, lines 527–550		
	**4**	Study aims, objectives, and hypotheses	p. 3, lines 96–100		
**METHODS**					
Study Design	**5**	Prospective or retrospective study	p. 3, lines 103–105		
	**6**	Study goal	pp. 3–4, lines 96–100, 132–133		
Data	**7**	Data sources	p. 3, lines 107–116; p. 15, lines 551–565 (partially reported)		
	**8**	Inclusion and exclusion criteria	p. 3, lines 107–116 (partially reported)		
	**9**	Data pre-processing	pp. 5–6, lines 178–212		
	**10**	Selection of data subsets	p. 3, lines 109–116; p. 5, lines 180–182		
	**11**	De-identification methods	p. 16, lines 627–628 (partially reported)		
	**12**	How missing data were handled	p. 3, lines 113–116; p. 7, lines 271–273 (partially reported)		
	**13**	Image acquisition protocol	p. 5, lines 178–184 (partially reported)		
Reference Standard	**14**	Definition of method(s) used to obtain reference standard	p. 5, lines 159–167		
	**15**	Rationale for choosing the reference standard	p. 2, lines 53–60; p. 5, lines 159–170 (partially reported)		
	**16**	Source of reference standard annotations	p. 5, lines 166–168 (partially reported)		
	**17**	Annotation of test set	p. 5, lines 159–167 (partially reported)		

	**18**	Measures of inter- and intra-rater variability of features described by the annotators	Not performed	**X**	
Data Partitions	**19**	How data were assigned to partitions	pp. 6–7, lines 228–230, 261–267 (partially reported)		
	**20**	Level at which partitions are disjoint	p. 6, lines 228–230		
Testing Data	**21**	Intended sample size	Not performed (formal sample-size rationale)	**X**	

Section / Topic	No.	Item	Page / Line	No	NA
Model	**22**	Detailed description of model	pp. 6–7, lines 214–225, 255–261 (partially reported)		
	**23**	Software libraries, frameworks, and packages	Not reported	**X**	
	**24**	Initialization of model parameters	p. 6, lines 218–225 (partially reported)		
Training	**25**	Details of training approach	pp. 6–7, lines 220–225, 261–267 (partially reported)		
	**26**	Method of selecting the final model	pp. 6–7, lines 226–228, 255–261; p. 10, lines 326–349		
	**27**	Ensembling techniques			**X**
Evaluation	**28**	Metrics of model performance	pp. 6–7, lines 230–232, 265–267; p. 15, lines 591–594 (decision-curve analysis not performed)		
	**29**	Statistical measures of significance and uncertainty	pp. 6–7, lines 240–245, 262–267 (partially reported)		
	**30**	Robustness or sensitivity analysis	p. 7, lines 261–267; p. 10, lines 333–340 (partially reported)		
	**31**	Methods for explainability or interpretability	p. 6, lines 232–235; p. 13, lines 454–467		
	**32**	Evaluation on internal data	pp. 6–7, lines 228–230, 261–267; p. 15, lines 586–590		
	**33**	Testing on external data	pp. 15–16, lines 586–599, 604–612 (external testing/validation not performed)		
	**34**	Clinical trial registration			**X**
**RESULTS**					
Data	**35**	Numbers of patients or examinations included and excluded	p. 3, lines 107–116; pp. 7–8, lines 271–299		
	**36**	Demographic and clinical characteristics of cases in each partition	p. 4, lines 138–153; pp. 7–8, lines 278–299 (partially reported; formal fairness assessment not performed)		
Model performance	**37**	Performance metrics and measures of statistical uncertainty	pp. 8–11, lines 303–369 (partially reported)		
	**38**	Estimates of diagnostic performance and their precision	pp. 8–11, lines 303–369; p. 15,		

			lines 591–594 (partially reported; calibration analysis not performed)		
	**39**	Failure analysis of incorrect results	Not performed	**X**	
**DISCUSSION**					
	**40**	Study limitations	p. 13, lines 454–467; p. 15, lines 551–599		
	**41**	Implications for practice, including intended use and/or clinical role	p. 14, lines 527–550; p. 16, lines 604–612		
**OTHER INFORMATION**					
	**42**	Provide a reference to the full study protocol or to additional technical details	Not reported	**X**	
	**43**	Statement about the availability of software, trained model, and/or data	p. 16, lines 629–633		
	**44**	Sources of funding and other support; role of funders	p. 16, line 623		

* Indicate page and/or line number for each checklist item that is present. NA = not applicable.
