# Supplementary material for: Artificial Intelligence-Based Prediction of Pancreatic Stone Clearance in Pancreatolithiasis Using Pretreatment CT Images and Clinical Features
Source: Diagnostics (Basel). 2026 Aug 24;16(17):2700. doi: 10.3390/diagnostics16172700 (PMC13564669; doi:10.3390/diagnostics16172700)
Supplement: Supplementary file 1 [file diagnostics-16-02700-s001.zip › Supplementary Table S1.pdf]

Table S1. Comparison of Patient Characteristics and Stone Clearance Outcomes According to the Availability of Extractable Pretreatment Noncontrast Abdominal CT Image Data

Characteristic	Extractable pretreatment non-contrast abdominal CT image data		
	Available, n (%) (n = 91)	Unavailable, n (%) (n = 104)	*P*
Age			
< 65 Years (n = 133)	56 (62)	77 (74)	0.086
≥ 65 Years (n = 62)	35 (38)	27 (26)	
Sex			
Male (n = 162)	78 (86)	84 (81)	0.467
Female (n = 33)	13 (14)	20 (19)	
Symptoms			
Present (n = 140)	52 (57)	88 (85)	<0.001
Absent (n = 55)	39 (43)	16 (15)	
Etiology			
Alcoholic (n = 126)	54 (59)	72 (69)	0.197
Non-alcoholic (n = 69)	37 (41)	32 (31)	
Number of Stones			
Single (n = 100)	41 (45)	59 (57)	0.138
Multiple (n = 95)	50 (55)	45 (43)	
Size of Stones			
≤ 14 mm (n = 140)	63 (69)	77 (74)	0.559
≥ 15 mm (n = 55)	28 (31)	27 (26)	
Distribution of Stones			
One Area (n = 164)	76 (84)	88 (85)	0.990
Two Areas or More (n = 31)	15 (16)	16 (15)	
Stricture of the Main Pancreatic Duct			
Present (n = 47)	13 (14)	34 (33)	0.005
Absent (n = 148)	78 (86)	70 (67)	
Stone Clearance			
Success (n = 146)	54 (59)	92 (88)	<0.001
Failure (n = 49)	37 (41)	12 (12)	
